# Selecting Core Outcomes for Randomised Effectiveness trials In Type 2 diabetes (SCORE-IT): a patient and healthcare professional consensus on a core outcome set for type 2 diabetes

**DOI:** 10.1136/bmjdrc-2019-000700

**Published:** 2019-12-29

**Authors:** Nicola L Harman, John P H Wilding, Dave Curry, James Harris, Jennifer Logue, R John Pemberton, Leigh Perreault, Gareth Thompson, Sean Tunis, Paula R Williamson, on behalf of the SCORE-IT Study Team, Serena Battaglia, Florence Bietrix, Jacques Demotes-Mainard, Valerie Gailus-Durner, Silvio Garattini, Tim Moser, Cecilia A C Prinsen, Michael Raess, Adrian Sanz-Moreno, Caroline B Terwee

**Affiliations:** 1 Department of Biostatistics, University of Liverpool, Liverpool, UK; 2 Institute of Ageing and Chronic Disease, University of Liverpool, Liverpool, UK; 3 University of Liverpool, Liverpool, UK; 4 Institute of Cardiovascular and Medical Sciences, University of Glasgow, Glasgow, UK; 5 Division of Endocrinology, Metabolism and Diabetes, University of Colorado, Denver, Colorado, USA; 6 Colorado School of Public Health, Aurora, Colorado, USA; 7 Center for Medical Technology Policy (CMTP), Baltimore, Maryland, USA

**Keywords:** Type 2 diabetes, randomized clinical trials, outcomes, outcomes research

## Abstract

**Objectives:**

Heterogeneity in outcomes measured across trials of glucose-lowering interventions for people with type 2 diabetes impacts on the ability to compare findings and may mean that the results have little importance to healthcare professionals and the patients that they care for. The SCORE-IT study (Selecting Core Outcomes for Randomised Effectiveness trials In Type 2 diabetes) has addressed this issue by establishing consensus on the most important outcomes for non-surgical interventions for hyperglycemia in type 2 diabetes.

**Research design and methods:**

A comprehensive list of outcomes was developed from registered clinical trials, online patient resources, qualitative literature and long-term studies in the field. This list was then scored in a two-round online Delphi survey completed by healthcare professionals, people with type 2 diabetes, researchers in the field and healthcare policymakers. The results of this online Delphi were discussed and ratified at a face-to-face consensus meeting.

**Results:**

173 people completed both rounds of the online survey (116 people with type 2 diabetes, 37 healthcare professionals, 14 researchers and 6 policymakers), 20 of these attended the consensus meeting (13 people with type 2 diabetes and 7 healthcare professionals). Consensus was reached on 18 core outcomes across five domains, which include outcomes related to diabetes care, quality of life and long-term diabetes-related complications.

**Conclusions:**

Implementation of the core outcome set in future trials will ensure that outcomes of importance to all stakeholders are measured and reported, enhancing the relevance of trial findings and facilitating the comparison of results across trials.

**Significance of this study**

**What is already known about this subject?**

- A systematic review of active clinical trials registered with ClinicalTrials.gov identified marked heterogeneity in the outcomes measured in trials of glucose-lowering interventions for people with type 2 diabetes.
- This inconsistency in outcomes impacts on the ability to compare findings and may mean that the results have little importance to healthcare professionals and the patients that they care for.

**What are the new findings?**

- Eighteen outcomes have been included in the SCORE-IT (Selecting Core Outcomes for Randomised Effectiveness trials In Type 2 diabetes) core outcome set, across five domains, which reflect outcomes related to diabetes care, quality of life and diabetes-related complications.
- This core outcome set has been developed with input from all stakeholders including people with type 2 diabetes, healthcare professionals, researchers in the field and healthcare policymakers/payers, and has ensured that all participants had an equal voice when deciding the most important outcomes.

**How might these results change the focus of research or clinical practice?**

- Implementation of the SCORE-IT core outcome set in future clinical trials, of glucose-lowering interventions, will increase the relevance of research to all stakeholders and will allow results from different trials to be more easily compared and combined.
- This increased potential for meta-analysis will enable new, effective treatments to be made available to people with type 2 diabetes more quickly.

## Background

Type 2 diabetes is a global pandemic. Current estimates indicate that 623 million people aged 20–79 will be affected by diabetes by 2045, with the majority of these cases being type 2 diabetes.^1–3^

Treatment for type 2 diabetes is targeted at the hyperglycemia arising due to a resistance to insulin action and an inadequate insulin secretory response.^4 5^ Lifestyle changes or pharmacotherapy aim to control blood glucose levels and avoid hyperglycemia and associated long-term complications,^6–12^ and these may also be supplemented with bariatric surgical intervention.^13^

A recent review of open (actively recruiting or in follow-up period), phase III and IV trials registered with ClinicalTrials.gov identified considerable variation in the outcomes measured and reported for glucose-lowering therapies in people with type 2 diabetes.^14^ This variation may impact on the ability to compare studies and hinder evidence synthesis, contributing to waste in research.^15^ Furthermore, of the outcomes measured in the included trials, only 10% represented patient-reported outcomes. It is possible to address these issues and to increase the relevance of research by developing a core outcome set (COS), representing an agreed standardized set of outcomes that should be measured and reported in all trials for a specific clinical area.^16^ To date only two studies have investigated important outcomes for diabetes. Byrne *et al* 17 have developed a COS for young adults with type 1 diabetes, and Murad *et al* 18 explored outcomes important to patients with diabetes using a single item on a questionnaire that ranked a list of 10 outcomes.

The aim of the SCORE-IT study (Selecting Core Outcomes for Randomised Effectiveness trials In Type 2 diabetes) was to address this gap in outcomes research and develop a COS for use in clinical trials of non-surgical therapeutic interventions for the treatment of hyperglycemia in adults with type 2 diabetes that includes input from all stakeholders.

## Methods

### Study overview

**Figure 1:**
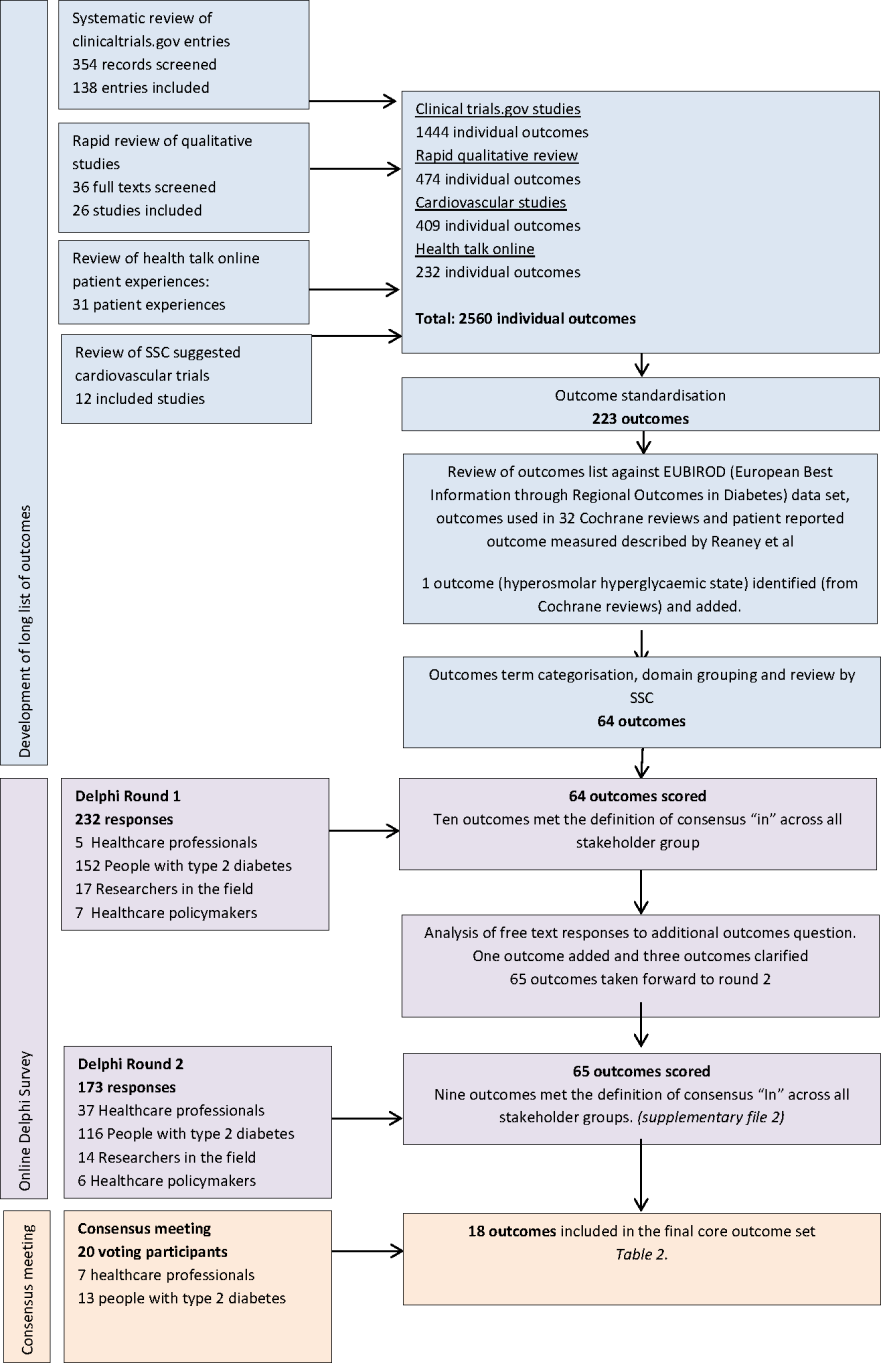
Core outcome set development overview. SSC, Study Steering Committee.

The development of the COS involved three stages (figure 1): (1) the generation of a long list of outcomes for use in an online Delphi; (2) a two-round online Delphi survey with key stakeholders; and (3) a face-to-face consensus meeting to discuss the results of the Delphi survey and agree on the COS. The methods for each step are described briefly below. A study protocol, systematic review and qualitative review describing methods in full have been published elsewhere.^14 19 20^

### Outcome list generation

The list of outcomes was generated from a number of sources^19^: a systematic review of open trials registered with ClinicalTrials.gov, a rapid review of the qualitative literature and extraction of outcomes from patient experiences reported on HealthTalk Online (www.healthtalk.org). The detailed search strategies for ClinicalTrials.gov and for the qualitative review have been published elsewhere.^14 20^ In addition to these sources, outcomes were extracted from transcripts of video clips of adults aged 18 years and over with type 2 diabetes who shared their experience on the publicly available HealthTalk online website.^21^ The Study Steering Committee (SSC) also provided a list of long-term cardiovascular outcome studies in people with type 2 diabetes from which outcomes were extracted.^22–33^ Outcomes were extracted verbatim, for each source, before being grouped using a standardized outcome name and categorized according to the taxonomy of Dodd *et al*.^34^ The list was cross-checked against outcomes and domains included in the patient-reported outcome measures (PROMs) identified by Reaney *et al*,^35^ the BIRO (Best Information through Regional Outcomes) common data set for diabetes (www.eubirod.eu) and relevant Cochrane reviews. To identify relevant reviews the Cochrane database of systematic reviews was searched for “type 2 diabetes” in the “title,” “abstract” and “key words” fields. The resulting list of outcomes was reviewed by the SSC and outcomes further grouped or excluded if measured in a single study and/or considered to be of limited clinical importance to glucose-lowering interventions. Each outcome was written using plain language and feedback sought from the public contributor members of the SSC on the acceptability and their understanding of the wording used.

### Online Delphi survey of stakeholders

The long list of outcomes was used to populate an online Delphi survey, delivered using the DelphiManager platform.^36^ Participants were invited from four key stakeholder groups: people with type 2 diabetes and their carers; healthcare professionals involved in delivering care for people with type 2 diabetes; researchers in the field; and healthcare policymakers/payers. Invitations to participate were shared with national and international patient and professional organizations, which were asked to distribute the invitations to their members (online supplementary file 1). We also approached the lead contact of the studies included in the ClinicalTrials.gov review, authors of relevant Cochrane reviews, researchers in receipt of funding from a large UK diabetes charity, and program leads at the National Institute of Diabetes and Digestive and Kidney Diseases of the National Institutes of Health and UK-based diabetes research centers. Finally, health professionals in the UK were contacted via the Wilmington’s UK database of health professionals. Policymakers were identified through the International Network of Agencies for Health Technology Assessment (INAHTA) members list and organizations approached individually.

10.1136/bmjdrc-2019-000700.supp1 Supplementary data

The Delphi process was completed using two rounds (termed R1 and R2). In each round participants were presented with the list of outcomes and asked to score each outcome on how important it was to include in the COS, using a 9-point Likert scale presented in formats 1–9, with 1–3 labeled “not important,” 4–6 labeled “important but not critical,” and 7–9 labeled “critically important.”^37^ At the end of R1, participants were invited to submit additional outcomes, and these outcomes were reviewed by the SSC and any suggestions representing a new outcome added to the list to be scored in R2. Outcomes were not removed from the list between R1 and R2.

During R2, participants were shown the distribution of scores for each stakeholder group for each outcome together with their own score from R1 and asked to score the outcome again, using the same 1–9 Likert scale, taking this information into consideration.

### Consensus meeting

A face-to-face consensus meeting was held in Liverpool, UK, and the results of the online Delphi survey were presented. Participants who had completed both R1 and R2 of the Delphi were invited to attend. Prior to the meeting, participants received a copy of their scores from the online survey and a consensus matrix detailing the results of R2 by stakeholder group (online supplementary file 2) and which outcomes had reached the predefined definition of consensus “in” or consensus “out” (table 1).

10.1136/bmjdrc-2019-000700.supp2 Supplementary data

**Table 1:** Definition of consensus

Consensus classification	Description	Definition
Consensus in	Consensus that outcome should be included in the core outcome set.	70% or more participants in each stakeholder group scoring as 7–9 and <15% participants in each stakeholder group scoring as 1–3.
Consensus out	Consensus that outcome should not be included in the core outcome set.	50% or fewer participants scoring 7–9 in each stakeholder group.
No consensus	Uncertainty about the importance of outcome.	Anything else.

The meeting was chaired by an independent non-clinical researcher with expertise in COS development methodology. Outcomes that had reached consensus “in” after R2 in all four stakeholder groups were presented first, followed by outcomes that had reached consensus “out” after R2 in all four stakeholder groups. Meeting participants were asked if they disagreed with the inclusion or exclusion of these items from the COS, respectively. All outcomes with two or more stakeholder groups reaching consensus “in” were discussed, outcomes with one stakeholder group reaching consensus “in” were shown to meeting participants, and participants asked if any of these should be discussed. Outcomes with no consensus and no group scoring consensus “in” were not discussed.

Views for and against inclusion in the COS were sought by the meeting chair, who also ensured that participants had equal opportunity to comment prior to voting. Voting took place anonymously using the TurningPoint software and handsets (Turning Technologies, Youngstown, USA). Following voting the results were displayed to participants by stakeholder group. For the purpose of the consensus meeting, stakeholder groups were condensed to healthcare professionals (this group included researchers in the field who also had a clinical role) and people with type 2 diabetes. Healthcare policymakers were present to provide their perspective and contribute to the discussions. The definition of consensus used in the Delphi survey (table 1) was applied with both groups required to reach the definition of consensus “in” for the outcome to be included in the COS. The final COS was presented at the end of the meeting and also included in a summary sent to participants after the meeting.

### Other analyses

Attrition bias between R1 and R2 of the online Delphi was assessed by comparing the distribution of mean R1 scores for participants completing R1 only and participants completing both R1 and R2. Satisfaction with the consensus meeting process, organization and outcome was assessed using a questionnaire (online supplementary file 3).

10.1136/bmjdrc-2019-000700.supp3 Supplementary data

### Study registration and study oversight

The SCORE-IT study was prospectively registered with the COMET Initiative (Core Outcome Measures in Effectiveness Trials).^38^ The SSC composition has been described previously.^19^ The SCORE-IT study is reported in line with the Core Outcome Set – Standards for Reporting reporting guidance.^39^

## Results

An overview of the SCORE-IT COS development process and final COS is shown in figure 1. The final COS includes 18 outcomes across five domains (table 2).

**Table 2:** Outcomes included in the SCORE-IT core outcome set

Outcome	Domain
Glycemic control: how well someone’s blood glucose is controlled.	Physiological/clinical
Global quality of life: someone’s overall quality of life, including physical, mental and social well-being.	Life impact
Activities of daily living: being able to complete usual everyday tasks and activities, including those related to personal care, household tasks or community-based tasks.	Life impact
Body weight: how much someone weighs.	Physiological/clinical
Kidney function: how well someone’s kidneys are working.	Physiological/clinical
Hyperglycemia: how often someone has high blood glucose.	Physiological/clinical
Hypoglycemia: how often someone has low blood glucose levels.	Physiological/clinical
Visual deterioration or blindness: if someone’s eyesight gets worse or if they have loss of vision including blindness.	Physiological/clinical
Neuropathy: damage to the nerves caused by high glucose. This can lead to tingling and pain or numbness in the feet or legs. It can also affect bowel control, stomach emptying and sexual function.	Physiological/clinical
Having gangrene or having an amputation of the leg, foot or toe.	Physiological/clinical
Non-fatal myocardial infarction: having a heart attack that is not fatal.	Physiological/clinical
Heart failure.	Physiological/clinical
Cerebrovascular disease, including stroke, subarachnoid hemorrhage, transient ischemic attack and vascular dementia.	Physiological/clinical
How often someone is admitted to hospital because of their diabetes.	Resource use
Hyperglycemic emergencies (to include diabetic ketoacidosis and hyperosmolar hyperglycemic state).	Physiological/clinical
Side effects of treatment: any unwanted effects of the treatment.	Adverse events
Overall survival: how long someone lives.	Death
Death from a diabetes-related cause, such as heart disease.	Death

SCORE-IT, Selecting Core Outcomes for Randomised Effectiveness trials In Type 2 diabetes.

### Development of the long list of outcomes

The systematic review of clinical trials and the rapid review of qualitative studies have been presented in detail elsewhere.^14^ The review of clinical trials yielded 1444 individual outcomes and the qualitative review 474. These were combined with 409 outcomes from the long-term cardiovascular outcome studies and 232 outcomes identified from HealthTalk Online. The resulting 2560 outcomes were reviewed and outcome names standardized to give 223 outcomes. These 223 outcomes were reviewed against the remaining data sources. One additional outcome “hyperosmolar hyperglycaemic state” was identified from the review of outcomes used in Cochrane reviews and added to the long list. No further outcomes were identified from the BIRO data set or review of PROMs.^35^

The 223 outcomes were mapped onto a 38-category system and grouped under five domains (mortality n=5, life impact n=67, physiological/clinical n=127, resource use n=22 and adverse events n=2).^34^ These outcomes were then presented to the SSC, and after discussion 64 outcomes (online supplementary file 4) were taken forward to the online Delphi.

10.1136/bmjdrc-2019-000700.supp4 Supplementary data

### Online Delphi process

One hundred and seventy three participants completed both R1 and R2 of the online survey. Participants comprised 37 healthcare professionals, 116 people with type 2 diabetes or their carers, 14 researchers in the field and 6 healthcare policymakers (table 3).

**Table 3:** Characteristics of Delphi participants completing round 1 and round 2

	n (%)
Healthcare professionals	37 (100)
Occupation	
Consultant	17 (21)
Dietitian	7 (9)
General practitioner	18 (23)
Pharmacist	2 (3)
Specialist nurse/practice nurse	36 (45)
Country of residence	
Austria	1 (3)
Germany	1 (3)
Greece	1 (3)
India	1 (3)
Mexico	1 (3)
Singapore	1 (3)
Switzerland	1 (3)
UK	30 (81)
People with type 2 diabetes and their carers	116 (100)
Age (years)	
30–39	3 (3)
40–49	8 (7)
50–59	19 (16)
60–69	55 (47)
70–79	29 (25)
>80	2 (2)
Country of residence	
Greece	1 (1)
UK	115 (99)
Researchers in the field	14 (100)
Country of residence	
Malaysia	2 (14)
Singapore	1 (7)
South Africa	1 (7)
UK	9 (64)
Not reported	1 (7)
Healthcare policymakers/payers	6 (100)
Country of residence	
Argentina	1 (17)
Australia	1 (17)
Austria	1 (17)
Canada	1 (17)
Germany	1 (17)
Sweden	1 (17)

At the end of R1, 10 outcomes met the predefined criteria for inclusion in the COS across all four stakeholder groups. Fifty-one responses were received to the free-text question asking participants if there were any additional outcomes they would like to add. These outcomes were reviewed by the SSC and one outcome “gut microbiome - the type/number of bacteria in someone’s digestive tract” was added and scored by participants in R2. A further three outcomes (activities of daily living, satisfaction with treatment and care, and emotional well-being) were modified based on the free-text response to clarify the outcome.

At the end of R2 of the Delphi, nine outcomes had reached consensus, for inclusion in the COS, across all four stakeholder groups, and nine outcomes had reached the definition for exclusion from the COS (online supplementary file 2).

Six outcomes reached the definition of “consensus in” in both R1 and R2 and have been included in the final COS.

The overall attrition rate between R1 and R2 was 25%, with the highest attrition rate observed for specialist/practice nurses (table 4).

**Table 4:** Attrition rates between rounds

Stakeholder	Registered, n (% of total registrations)	Withdrawn prior to completing R1	Withdrawn after R1 and before R2	Completed R1, n (% of registrations minus withdrawals before R1)	Completed R2, n (% of R1)
Healthcare professionals	80 (25)	0	0	56 (70)	37 (66)
Consultant	17 (5)	0	0	10 (59)	8 (80)
Dietitian	7 (2)	0		6 (86)	5 (83)
General practitioner	18 (6)	0	0	13 (72)	8 (62)
Pharmacist	2 (1)	0	0	2 (100)	2 (100)
Specialist/practice nurse	36 (11)	0	0	25 (69)	14 (56)
Researchers in the field	20 (6)	0	1	17 (85)	14 (88)
Policymakers/payers	9 (3)	0	0	7 (78)	6 (86)
People with type 2 diabetes or their carers	211 (66)	5	2	153 (74)	116 (77)
Carer	5 (2)	0	0	3 (60)	1 (33)
Patient	206 (64)	5	2	150 (75)	115 (78)
Total	320	5	3	233 (74)	173 (75)

R1, round 1; R2, round 2.

The impact of attrition between rounds was assessed by comparing the average R1 scores of those who did not complete R2 against the distribution of scores for those completing both R1 and R2. Overall the distribution of average scores of those who did not complete R2 was similar to those completing both R1 and R2 for all stakeholder groups, suggesting that attrition bias was not present between rounds (online supplementary file 5).

10.1136/bmjdrc-2019-000700.supp5 Supplementary data

### Consensus meeting

Twenty participants attended the consensus meeting (7 healthcare professionals and 13 people with type 2 diabetes); in addition to the 20 voting participants, there were 3 healthcare policymakers/payers who contributed to discussion along with members of the SSC. In the consensus meeting a further nine outcomes met the definition for inclusion in the COS, in addition to the nine outcomes that had reached the definition of consensus for inclusion at the end of R2 of the Delphi (online supplementary file 6). Of these outcomes, eight required further discussion by the SSC at a follow-up teleconference (online supplementary file 7). In addition to wording changes, one outcome “diabetic ketoacidosis” was amended to “hyperglycaemic emergencies (to include diabetic ketoacidosis and hyperosmolar hyperglycaemic state).” The SSC also reflected on the outcomes “hyperglycaemia,” how this had been interpreted by Delphi participants and that further discussion/think aloud work prior to launching the Delphi may have been needed. Finally, the SSC discussed the comment raised at the consensus meeting to add “prolongation of hospital stay” to the outcome “how often someone is admitted to hospital because of their diabetes.” All agreed that this was a separate outcome that had not been scored or added in the Delphi but is an important point for future discussion.

10.1136/bmjdrc-2019-000700.supp6 Supplementary data

10.1136/bmjdrc-2019-000700.supp7 Supplementary data

Feedback forms from the meeting were completed by 4 (57%) healthcare professionals and 12 (92%) people with type 2 diabetes. All participants were satisfied with the way the meeting was facilitated, felt able to contribute to the meeting and felt comfortable expressing their views. In terms of the consensus meeting producing a fair result, one health professional felt that voting may have been influenced by a dominant participant, and one person with type 2 diabetes neither agreed nor disagreed with the statement.

## Discussion

The SCORE-IT study has developed patient and health professional consensus on outcomes for trials of the treatment of hyperglycemia in people with type 2 diabetes. The process to achieve consensus has ensured that all stakeholders including people with type 2 diabetes, healthcare professionals, researchers in the field and healthcare policymakers/payers have been able to contribute to the final COS. We recommend that future trials of interventions to treat hyperglycemia in people with type 2 diabetes use the SCORE-IT COS. This COS does not prevent other outcomes being measured, as appropriate to the intervention, but rather represents the minimum that should be measured.

Particular strengths of the SCORE-IT COS include the use of methods meeting the COS-STAD (Core Outcome Set - STAndards for Development) recommendations,^40^ published in a study protocol prior to undertaking the study.^19^ This study has also engaged multiple stakeholder groups including health professionals and people with type 2 diabetes to achieve consensus on the most important outcomes. Only 3% of COS to date have included input from healthcare policymakers,^41^ the inclusion of policymakers in the present study has ensured that important outcomes used when evaluating the available evidence and making decisions are taken into consideration in the final COS. In the SCORE-IT study members of the International Network of Agencies for Health Technology Assessment (INAHTA) were approached with an invitation to take part. Of the 50 members, six (12%) completed R1 and R2 suggesting that further work is needed to engage with HTA organisations to facilitate the contribution of this stakeholder group to COS development.

Although this study has had some international input, engagement both in the UK and on an international level was challenging, with only one patient and a fifth of healthcare professionals and researchers combined based outside of the UK. We sought to improve international input from people with type 2 diabetes through engagement with patient organizations and translation of the Delphi into the appropriate local language. However, despite Polish and Brazilian Portuguese versions being distributed, via direct email to members of patient organizations, participation was low, with only one person completing R1 of the Delphi in Polish. Choosing appropriate outcomes to measure is a top methodological priority for trialists working in low-income and middle-income countries (LMICs).^42^ A recent review found the number of COS being developed in some LMICs has increased,^41^ yet the number of participants in each COS, the methods of engagement and the source of recruitment have yet to be explored. Further work is warranted in the field of COS research more generally on how best to engage stakeholders and facilitate participation nationally, internationally and particularly in countries where representation in the COS development process is low. For the SCORE-IT COS it will be important to evaluate the acceptability of the current COS to patients and professionals in other countries, particularly where healthcare systems differ from that in the UK.

The SCORE-IT COS is specific for type 2 diabetes, yet there is overlap with outcomes of importance to young adults with type 1 diabetes identified by Byrne *et al*.^17^ Of the eight outcomes in their COS, all three outcomes that are physiological/clinical are included in the current SCORE-IT COS.^17^ Quality of life is also common across the two COS, although in the study by Byrne *et al* 17 this was amended to “diabetes related quality of life” in response to discussion at the consensus meeting. Other outcomes included in the COS for young adults with type 1 diabetes were included in the long list of outcomes scored in R1 of the present study, yet these outcomes did not reach the definition of consensus “in.” Self-management behavior, specifically “Diabetes self-care activities…”, was discussed further at the consensus meeting but did not reach the definition of consensus for inclusion in the COS. The SCORE-IT COS includes additional outcomes that reflect complications of hyperglycemia, which were not included in the COS developed by Byrne *et al*, suggesting that while there are some similarities the priorities of the stakeholders vary depending on the type of diabetes and the age group of participants.

Murad *et al* 18 included participants with both type 1 (5%) and type 2 (93%) in a survey to identify participants’ top 5 outcomes from a list of 10. All 10 of the outcomes ranked were included in the current Delphi survey, and all, with the exception of the need for photocoagulation, scored in the present study as retinopathy, were included in the current COS, with hemoglobin A1c and end-stage renal disease included in the “glycaemic control” and “kidney function” outcomes, respectively. The list of outcomes used by Murad *et al* 18 was generated from a panel of eight patients and ranked by patients only. Our approach to the development of the outcomes list and engagement of people with type 2 diabetes, healthcare professionals and researchers have identified an additional 10 outcomes, including outcomes within the life impact domain (quality of life and activities of daily living), which are important to all stakeholders.

The International Consortium for Health Outcomes Measurement (ICHOM) has recently reported a standard set for diabetes in adults. The development of this standard set has included international input from 26 experts (3 patients and 23 clinicians) and includes 26 outcomes. Fifty-four percent of the ICHOM standard set is reflected in the SCORE-IT COS, with some subtle differences. Most notably the life impact outcomes between the two outcome sets differ. Psychological well-being, diabetes distress and depression are included in the ICHOM, while these outcomes, scored collectively in SCORE-IT as “Emotional wellbeing,” did not reach the definition of “consensus in” in any round of the Delphi survey. Instead participants of the SCORE-IT study rated “global quality of life” and “activities of daily living” as the most important life impact outcomes. Two outcomes in the ICHOM standard set, “periodontal health” and “emergency room utilisation,” were not included in the SCORE-IT Delphi list of outcomes. Periodontal health was not measured/identified from any of the sources used to generate the long list of outcomes, while the need to attend the emergency room was identified in the systematic review but measured only in a single study and not taken forward to the Delphi survey. Neither outcome was added to the list by Delphi participants completing R1 of the Delphi survey.

While there is substantial overlap between the ICHOM standard set and the SCORE-IT COS, differences may reflect the scope of the projects, clinical practice versus clinical trials, respectively, and may also be influenced by the methods used and the type, number and geographic location of the stakeholders involved. Nevertheless the overlap between studies is positive, and if outcomes are captured routinely in clinical practice then this may help improve the efficiency of clinical trials and reduce the burden to trial participants.

## Conclusions

The COS developed in the SCORE-IT study can be applied to future clinical trials of non-surgical interventions to treat hyperglycemia, and its use will allow comparisons to take place across trials, thereby reducing waste in research. The next steps will include seeking consensus on how these outcomes should be measured and to provide this guidance to researchers.
