# Supplementary material for: Selecting Core Outcomes for Randomised Effectiveness trials In Type 2 diabetes (SCORE-IT): a patient and healthcare professional consensus on a core outcome set for type 2 diabetes
Source: BMJ Open Diabetes Res Care. 2019 Dec 29;7(1):e000700. doi: 10.1136/bmjdrc-2019-000700 (PMC6936506; doi:10.1136/bmjdrc-2019-000700)
Supplement: Supplementary data [file bmjdrc-2019-000700supp001.pdf]

Supplementary file 1. Organisations who distributed SCORE-IT study information for the online Delphi survey.

Organisation	Coverage	Scope	Dissemination activities
Diabetes UK	UK	People with type 2 diabetes, health professionals and researchers	Included on the organisations webpage "take part in research". https://www.diabetes.org.uk/research/take-part-in-research
			tweeted via the organisation's twitter account https://twitter.com/DUK_research/status/993845923095687168”
			Email sent to members of the 2019 professional conference organising committee.
European Association for the study of diabetes (EASD)	Europe	Health professionals and researchers	included on the organisation's webpage
British Dietetic Association	UK	Health professionals	Information included in the diabetes specialist group newsletter.
			Added to diabetes specialist group's private Facebook group
Association of British Clinical Diabetologists	UK	Health professionals	Study information added to website and also emailed directly to members
Research for the Future	UK	People with type 2 diabetes	Direct email sent to members
EURADIA (Alliance for European Diabetes Research)	Europe	Health professionals and researchers	Direct email sent to members
FEND (Foundation for European Nurses in Diabetes)	Europe	Health professionals	Direct email sent to members
Royal College of GPs Clinical Research and Innovation Centre	UK	Health professionals	Included in a newsletter emailed to members
NICE (National Institute for Clinical Excellence)	UK	Policymakers	emailed to type 2 diabetes committee members
Diabetes Ireland	Ireland	People with type 2 diabetes, health professionals and researchers	Included on organisations website and tweeted via organisation's twitter account
The Polish Diabetes Association	Poland	People with type 2 diabetes	Facilitated translation of the survey and emailed information directly to members
FENAD (Federação Nacional de Associações e Entitades de Diabetes)	Brazil	People with type 2 diabetes, health professionals and researchers	Translated study information emailed directly to members
WISDEM (Warwickshire Institute for the Study of Diabetes, Endocrinology and Metabolism)	UK	Researchers in the field	Email sent to team members
**Diabetes UK Local Groups**			
Croydon	UK	People with type 2 diabetes	Direct email sent to group members

Newport	UK	People with type 2 diabetes	Included in agenda for monthly meetings
Tunbridge Wells, Tonbridge and Sevenoaks	UK	People with type 2 diabetes	Discussed at a face to face meeting, study flyer and information sent to members by email
Burton	UK	People with type 2 diabetes	Study flyer distributed at a research day
West Lothian	UK	People with type 2 diabetes	Study information posted on group's public and private Facebook pages and information distributed at a face to face meeting
Nailsea Backwell and District	UK	People with type 2 diabetes	Study information emailed directly to members
Harrow and District	UK	People with type 2 diabetes	Study information emailed directly to members
Mansfield	UK	People with type 2 diabetes	Study flyers distributed at a face to face meeting
Cardigan Diabetes	UK	People with type 2 diabetes	Information distributed to group members by a specialist nurse
West Dorset	UK	People with type 2 diabetes	Study flyers distributed at a face to face meeting
Shrewsbury & District	UK	People with type 2 diabetes	Study flyers distributed at a face to face meeting
Stonehenge	UK	People with type 2 diabetes	Study flyers distributed at a face to face meeting
Basingstoke	UK	People with type 2 diabetes	Study information posted on the group's Facebook page
Exeter	UK	People with type 2 diabetes	Study information posted on the group's Facebook page
Lambeth and Southwark	UK	People with type 2 diabetes	Study flyers distributed at a face to face meeting
North Norfolk	UK	People with type 2 diabetes	Study flyers distributed at a face to face meeting
North Staffs Local Group	UK	People with type 2 diabetes	Included in newsletter and meeting agenda
Southampton and District	UK	People with type 2 diabetes	Study flyers distributed at a face to face meeting
Ilford and District	UK	People with type 2 diabetes	Study flyers distributed at a face to face meeting
Watford	UK	People with type 2 diabetes	Study flyers distributed at a face to face meeting also posted on group's Facebook page
York Adult	UK	People with type 2 diabetes	Study flyers distributed at a face to face meeting also posted on group's Facebook page
South Devon	UK	People with type 2 diabetes	Flyers sent for distribution at group meeting 7th July
Broadland and Waveney	UK	People with type 2 diabetes	Study flyers distributed at a face to face meeting
Chippenham District	UK	People with type 2 diabetes	Study flyers distributed at a face to face meeting
Chelmsford	UK	People with type 2 diabetes	Study information emailed to members and flyers distributed at a meeting
Herefordshire	UK	People with type 2 diabetes	Study flyers distributed at a face to face meeting also posted on group's Facebook page
Lincolnshire Project	UK	People with type 2	Study flyers distributed at a living with diabetes day

		diabetes	
