# Supplementary material for: Selecting Core Outcomes for Randomised Effectiveness trials In Type 2 diabetes (SCORE-IT): a patient and healthcare professional consensus on a core outcome set for type 2 diabetes
Source: BMJ Open Diabetes Res Care. 2019 Dec 29;7(1):e000700. doi: 10.1136/bmjdrc-2019-000700 (PMC6936506; doi:10.1136/bmjdrc-2019-000700)
Supplement: Supplementary data [file bmjdrc-2019-000700supp002.pdf]

Consensus Reached (9 outcomes).

Consensus 'In' (>= 70% 7-9 AND <=15% 1-3 in all stakeholder groups)

The following outcomes meet the study criteria for inclusion in the core outcome set.

In all groups of people who took part in both rounds of the online survey more than 70% gave a score of 7-9 to indicate that it was critical that this outcome be included in the core outcome set. Including these outcomes in the core outcome set means that they should be measured in every future study of glucose lowering interventions for people with type 2 diabetes.

			Healthcare Professionals (HCP)				Researchers in the field				People with type 2 diabetes				Healthcare Policymakers			
#	Domain	Outcome name	1-3	4-6	7-9	HCP result	1-3	4-6	7-9	Researcher Result	1-3	4-6	7-9	People with type 2 results	1-3	4-6	7-9	Policymak-er result
2	Death	Overall survival - how long someone lives	0	21	79	In	0	29	71	In	0	15	85	In	0	0	100	In
4	Death	Death from a specific cause such as heart disease	0	19	81	In	0	19	81	In	2	10	88	In	0	17	83	In
27	Physiological/ clinical	Heart failure	0	13	87	In	13	7	80	In	0	3	97	In	0	17	83	In
40	Physiological/ clinical	Having gangrene or having an amputation of the leg; foot or toe	0	3	97	In	14	7	79	In	0	3	97	In	0	0	100	In
43	Physiological/ clinical	Diabetic ketoacidosis- Diabetic ketoacidosis occurs if the body cannot produce enough insulin. It is a serious short term complica-tion of diabetes which can result in coma or even death if it is not treated quickly.	0	5	95	In	0	21	79	In	0	2	98	In	0	0	100	In
46	Physiological/ clinical	Hyperglycaemia - how often someone has high blood glucose	0	22	78	In	0	21	79	In	0	19	81	In	0	0	100	In
48	Physiological/ clinical	Hypoglycaemia - how often someone has low blood glucose levels.	0	5	95	In	0	0	100	In	1	9	90	In	0	0	100	In
58	Physiological/ clinical	Cerebrovascular disease (including stroke; subarachnoid haemorrhage; transient is-chaemic attack and vascular dementia	0	3	97	In	0	15	85	In	0	3	97	In	0	0	100	In
62	Resource use	How often someone is admitted to hospital because of their diabetes.	0	14	86	In	0	7	93	In	1	23	76	In	0	17	83	In

Consensus Reached (10 outcomes).

Consensus 'out' (<= 50% 7-9 in all stakeholder groups)

The following outcomes meet the study criteria for exclusion from the core outcome set. In all groups of people who took part less than 50% gave a score of 7-9 to indicate that it was critical that this outcome be included in the core outcome set.

This does not mean that these outcomes aren't important and that they should never be measured. Instead that they do not need to be measured by every study for type 2 diabetes.

			Healthcare Professionals (HCP)				Researchers in the field				People with type 2 diabetes				Healthcare Policymakers			
#	Domain	Outcome name	%1-3	%4-6	%7-9	HCP result	%1-3	%4-6	%7-9	result	%1-3	%4-6	%7-9	result	%1-3	%4-6	%7-9	result
16	Life impact	Support - The support that is available to someone from their family; friends; peers and workplace	8	61	32	low	13	69	19	low	7	60	33	low	50	50	0	low
20	Life impact	Sexual function - how well someone functions sexually	0	76	24	low	20	47	33	low	10	60	30	low	0	67	33	low
34	Physiological/ clinical	Body fat distribution - where body fat is stored in the body	5	74	21	low	14	57	29	low	4	50	45	low	67	33	0	low
41	Physiological/ clinical	Genital fungal infection- Having genital thrush or a similar infection	8	68	24	low	23	38	38	low	3	47	49	low	0	50	50	Low
51	Physiological/ clinical	Metabolic rate - The rate that the body uses energy	8	76	16	low	15	54	31	low	2	66	33	low	83	17	0	low
52	Physiological/ clinical	Appetite	5	81	14	low	29	50	21	low	6	77	17	low	33	67	0	low
53	Physiological/ clinical	Dietary intake - a measure of what someone has eaten	5	54	41	low	15	62	23	low	7	73	20	low	33	50	17	low
61	Resource use	All other costs of diabetes care apart from how often someone needs to see a healthcare professional.	0	65	35	low	0	69	31	low	2	73	25	low	17	67	17	low
64	Resource use	The impact that having diabetes has on the time or finances of others	5	73	22	low	14	57	29	low	7	69	24	low	17	67	17	low
65	Physiological/ clinical	Gut microbiome - the type/number of bacteria in someone's digestive tract.	19	68	14	low	25	50	25	low	6	59	35	low	83	17	0	low

No Consensus

Importance rated differently by different stakeholder groups, at least one group scoring "in" (32 outcomes)

			Healthcare Professionals (HCP)				Researchers in the field				People with type 2 diabetes				Healthcare Policymakers			
#	Domain	Outcome name	%1-3	%4-6	%7-9	HCP result	%1-3	%4-6	%7-9	Result	%1-3	%4-6	%7-9	result	%1-3	%4-6	%7-9	result
1	Adverse events	Side effects of treatment- any unwanted effects of the treatment	0	23	77	In	0	29	71	In	2	38	59	Medium	0	0	100	In
3	Death	Death from a medical procedure	0	17	83	In	0	25	75	In	3	8	89	In	0	33	67	Medium
8	Life impact	Need for change in treatment or having to start taking a new or additional treatment because of high blood glucose.	3	18	80	In	0	27	73	In	1	18	81	In	0	33	67	Medium
9	Life impact	Adherence to treatment - how well someone follows treatment instructions for example; taking all of their prescribed medications	0	18	83	In	0	19	81	In	0	11	89	In	17	17	67	Medium
12	Life impact	Global quality of life - someone's overall quality of life including physical; mental; and social wellbeing.	0	18	82	In	0	31	69	Medium	1	20	79	In	0	17	83	In
25	Physiological/ clinical	Cardiac function - how well the heart is working	0	26	74	In	7	43	50	Low	2	6	92	In	17	0	83	In
29	Physiological/ clinical	Nonfatal myocardial infarction - having a heart attack that is not fatal	0	16	84	In	14	21	64	Medium	1	7	92	In	0	17	83	In
30	Physiological/ clinical	Retinopathy - damage to the blood vessels in the back of the eye caused by high blood glucose levels	0	5	95	In	0	40	60	Medium	0	9	91	In	0	0	100	In
31	Physiological/ clinical	Visual deterioration or blindness - if someone's eyesight gets worse or if they have loss of vision including blindness	0	13	87	In	0	33	67	Medium	0	3	97	In	0	0	100	In
38	Physiological/ clinical	Having an infection in one or both feet including a foot ulcer; infection of the tissue	0	11	89	In	15	15	69	Medium	1	11	88	In	0	0	100	In
39	Physiological/ clinical	Having a bone infection (osteomyelitis). People with diabetes are more at risk of osteomyelitis especially if they have a foot ulcer.	0	16	84	In	25	25	50	Low	1	12	87	In	0	0	100	In

No Consensus continued *Importance rated differently by different stakeholder groups, at least one group scoring "in"*

			Healthcare Professionals (HCP)				Researchers in the field				People with type 2 diabetes				Healthcare Policymakers			
#	Domain	Outcome name	%1-3	%4-6	%7-9	HCP result	%1-3	%4-6	%7-9	Result	%1-3	%4-6	%7-9	result	%1-3	%4-6	%7-9	result
44	Physiological / clinical	Glycaemic control - how well someone's blood glucose is controlled.	0	8	92	In	0	0	100	In	0	6	94	In	0	50	50	Low
47	Physiological / clinical	Hyperosmolar hyperglycaemic state - a rare but serious and potentially life threatening complication of having very high blood glucose levels (often over 40mmol/L).	0	3	97	In	7	21	71	In	0	8	92	In	17	17	67	Medium
54	Physiological / clinical	Neuropathy - damage to the nerves caused by high glucose. This can lead to tingling and pain or numbness in the feet or legs. It can also affect bowel control; stomach emptying and sexual function	0	14	86	In	7	29	64	Medium	0	13	87	In	0	0	100	In
56	Physiological / clinical	Kidney function - how well someone's kidneys are working	0	19	81	In	0	21	79	In	0	9	91	In	0	50	50	Low
57	Physiological / clinical	Blood Pressure	0	16	84	In	0	8	92	In	0	20	80	In	33	17	50	Low
60	Physiological / clinical	Risk of developing cardiovascular disease (including lipid and lipoprotein markers of risk like cholesterol and triglycerides)	0	19	81	In	0	7	93	In	1	13	86	In	17	50	33	low
21	Life impact	Activities of daily living - being able to complete usual everyday tasks and activities including those related to personal care; house hold tasks or community based	0	29	71	In	6	44	50	low	1	23	76	In	0	33	67	Medium
59	Physiological / clinical	Peripheral vascular function - How well(veins and arteries) in the body (outside the heart) are working. Narrowing of these blood vessels; particularly in the legs; that can lead to pain; gangrene and amputation.	0	16	84	In	17	17	67	Medium	0	3	97	In	0	33	67	Medium
32	Physiological / clinical	Gastroparesis - this means that the stomach can't empty itself in the normal way. Symptoms can include feeling full/bloated; nausea/vomiting; loss of appetite; tummy pain/discomfort.	0	21	79	In	23	46	31	low	0	26	74	In	0	33	67	Medium
5	Life impact	Cognitive function - things about someone's memory; concentration; language; thinking and ability to understand instructions.	0	34	66	Medium	7	60	33	low	0	20	80	In	0	67	33	low

No Consensus continued

Importance rated differently by different stakeholder groups, at least one group scoring "in"

#	Domain	Outcome name	Healthcare Professionals (HCP) %1-3	%4-6	%7-9	HCP result	Researchers in the field %1-3	%4-6	%7-9	Result	People with type 2 diabetes %1-3	%4-6	%7-9	result	Healthcare Policymakers %1-3	%4-6	%7-9	result
6	Life impact	Applying diabetes knowledge - how well someone applies their diabetes knowledge to their care	0	51	49	low	0	69	31	low	1	29	70	In	17	67	17	low
13	Life impact	Discontinuation or dose reduction - if someone is able to stop taking or reduce the dose of all or some of their diabetes medications	5	33	62	Medium	0	27	73	In	1	46	53	Medium	17	50	33	low
18	Life impact	Diabetes self care activities- how well someone takes care of themselves in relation to their diabetes; for example; following dietary advice; foot care; testing and blood glucose levels	0	45	55	Medium	0	56	44	low	0	17	83	In	0	60	40	low
19	Life impact	Physical function - How well someone is able to function physically including how mobile they are and their physical strength.	0	39	61	Medium	0	60	40	low	2	24	74	In	0	33	67	Medium
26	Physiological / clinical	Cardiorespiratory fitness - how well your respiratory and circulatory systems respond to exercise	3	42	55	Medium	14	50	36	low	1	21	78	In	17	50	33	low
33	Physiological / clinical	Exocrine pancreas function -how well your pancreas functions to make enzymes that help digest food	8	58	34	low	31	31	38	low	0	23	77	In	33	50	17	low
35	Physiological / clinical	Body weight - how much someone weighs	0	24	76	In	0	43	57	Medium	1	37	62	Medium	17	33	50	low
36	Physiological / clinical	Liver function - how well the liver is working. The liver breaks down drugs and harmful substances. It also produces nutrients for the body to use including the storage and release of glucose.	0	39	61	Medium	15	15	69	Medium	0	8	92	In	33	33	33	low
45	Physiological / clinical	Glycaemic variability - how much someone's blood glucose changes over the course of the day	0	22	78	In	0	50	50	Low	2	30	68	Medium	17	33	50	Low
49	Physiological / clinical	Insulin sensitivity- how sensitive someone's body is to the effects of insulin	5	43	51	Medium	0	43	57	Medium	1	20	79	In	17	83	0	low
50	Physiological / clinical	Insulin secretion - how well the body responds to a meal	5	46	49	low	0	54	46	low	0	26	74	In	33	67	0	low

No Consensus: *Importance rated differently by different stakeholder groups - no group scored the importance as high (14 outcomes)*

			Healthcare Professionals (HCP)				Researchers in the field				People with type 2 diabetes				Healthcare Policymakers			
#	Domain	Outcome name	%1-3	%4-6	%7-9	HCP result	%1-3	%4-6	%7-9	Result	%1-3	%4-6	%7-9	result	%1-3	%4-6	%7-9	result
7	Life impact	Satisfaction with treatment and care - how satisfied someone with diabetes is with the treatments they are taking/following; the care that they receive from healthcare professionals and the amount and type of diabetes information available	3	40	58	Medium	0	56	44	low	0	37	63	Medium	17	67	17	low
11	Life impact	Fatigue - an overwhelming; sustained feeling of exhaustion; mental or physical tiredness; having little energy for physical and mental work.	3	36	62	Medium	13	50	38	low	2	37	61	Medium	17	33	50	Low
14	Life impact	Concomitant medication - how often and how many types of other medication someone has to take for example; blood pressure medication	5	56	38	low	0	47	53	Medium	3	48	50	low	17	67	17	low
15	Life impact	Perceived blood glucose control - how well someone with diabetes thinks their blood glucose is controlled	8	59	33	low	13	50	38	low	3	36	61	Medium	33	67	0	low
17	Life impact	Financial burden- The impact of someone's diabetes on their personal finances	0	42	58	Medium	13	44	44	low	7	55	38	low	33	50	17	low
22	Life impact	Impact of diabetes on work or ability to work	0	42	58	Medium	0	81	19	low	2	29	69	Medium	17	33	50	Low
23	Life impact	Being able to manage family responsibilities	5	39	55	Medium	13	69	19	low	3	37	60	Medium	0	83	17	low
24	Life impact	Social functioning - how able someone feels to join in social activities and maintain relationships with others.	3	50	47	low	19	56	25	low	3	60	37	low	0	33	67	Medium
28	Physiological/ clinical	Heart rate	13	61	26	low	29	57	14	low	1	36	63	Medium	33	50	17	low
37	Physiological/ clinical	Biomarkers of inflammation- Inflammation is the body's immune response to things like bacteria and viruses. The body can also sometimes attack its own tissues causing inflammation.	5	70	24	low	17	25	58	Medium	3	28	68	Medium	33	67	0	low
42	Physiological/ clinical	Urinary tract infection - having an infection in the urinary tract; including bladder; urethra or kidneys.	3	70	27	low	23	38	38	low	2	36	62	Medium	0	67	33	low
55	Physiological/ clinical	General health	0	42	58	Medium	0	57	43	low	0	32	68	Medium	0	50	50	Medium
63	Resource use	Healthcare resource utilisation - how often someone needs to see a healthcare professional	0	57	43	low	0	54	46	low	0	54	46	low	17	17	67	Medium
10	Life impact	Emotional wellbeing - emotional wellbeing includes lots of things like someone's mood; how often they worry; feel anxious or sad; how often they get angry or upset and their self esteem.	3	35	63	Medium	0	44	56	Medium	3	43	54	Medium	0	50	50	low
