# Supplementary material for: Selecting Core Outcomes for Randomised Effectiveness trials In Type 2 diabetes (SCORE-IT): a patient and healthcare professional consensus on a core outcome set for type 2 diabetes
Source: BMJ Open Diabetes Res Care. 2019 Dec 29;7(1):e000700. doi: 10.1136/bmjdrc-2019-000700 (PMC6936506; doi:10.1136/bmjdrc-2019-000700)
Supplement: Supplementary data [file bmjdrc-2019-000700supp003.pdf]

SCORE-IT Study consensus meeting evaluation form

Thank you very much for attending the SCORE-IT consensus meeting on the 24th January 2019.

We would value your feedback about the consensus meeting, to help improve future core outcome set work. If you could take a few moments to let us know your thoughts, it would be much appreciated

1. Please choose the option which describes you best:

☐Health care professional ☐Someone with type 2 diabetes ☐ Other

2.Please tell us how much you agree with each of the following statements:

The information that the organisers provided me with in advance of the meeting was helpful.

Strongly disagree	Disagree	Neither agree or disagree	Agree	Strongly agree
☐	☐	☐	☐	☐

Comments

I was satisfied with the process used to agree the core outcomes set on the meeting day.

Strongly disagree	Disagree	Neither agree or disagree	Agree	Strongly agree
☐	☐	☐	☐	☐

Comments

I was satisfied with the way the meeting was facilitated.

Strongly disagree	Disagree	Neither agree or disagree	Agree	Strongly agree
☐	☐	☐	☐	☐

Comments

I felt able to contribute to the meeting.

Strongly disagree	Disagree	Neither agree or disagree	Agree	Strongly agree
☐	☐	☐	☐	☐

Comments

I felt comfortable in communicating my views.

Strongly disagree	Disagree	Neither agree or disagree	Agree	Strongly agree
☐	☐	☐	☐	☐

Comments

The workshop produced a fair result.

Strongly disagree	Disagree	Neither agree or disagree	Agree	Strongly agree
☐	☐	☐	☐	☐

Comments

Please turn over...

SCORE-IT Study consensus meeting evaluation form

8 Do you have any comments about the practical arrangements for the workshop (e.g. venue, timing of the meeting, catering, number of breaks, or anything else)?

10. Was there anything else that could have been done to improve the workshop?

Thank you for your feedback

This form is based on the evaluation form developed with the COMPACTERS COS Study team (Steven Maclennan, Thomas Lam, Linda Pennet, Paula Williamson) and Heather Bagley (COMET) and was adapted from a previous evaluation used by the James Lind Alliance Mesothelioma Priority Setting Partnership Workshop) The form was further developed with the input of Bridget Young and Rosemary Humphreys (co-chairs of the COMET PoPPIE Working Group).
