# Supplementary material for: Selecting Core Outcomes for Randomised Effectiveness trials In Type 2 diabetes (SCORE-IT): a patient and healthcare professional consensus on a core outcome set for type 2 diabetes
Source: BMJ Open Diabetes Res Care. 2019 Dec 29;7(1):e000700. doi: 10.1136/bmjdrc-2019-000700 (PMC6936506; doi:10.1136/bmjdrc-2019-000700)
Supplement: Supplementary data [file bmjdrc-2019-000700supp004.pdf]

Supplementary file 4. List of outcomes scored in the online Delphi

Outcome	Domain
Side effects of treatment- any unwanted effects of the treatment	Adverse events
Overall survival - how long someone lives	Death
Death from a medical procedure	Death
Death from a specific cause such as heart disease	Death
Cognitive function - things about someone's memory, concentration, language and thinking	Life impact
Applying diabetes knowledge - how well someone applies their diabetes knowledge to their care	Life impact
Satisfaction with treatment and care - how satisfied someone with diabetes is with the treatments they are taking/following, the care that they receive from healthcare professionals, the amount, type and quality of diabetes information **and structured learning courses available .***	Life impact
Need for change in treatment or having to start taking a new or additional treatment because of high blood glucose.	Life impact
Adherence to treatment - how well someone follows treatment instructions for example, taking all of their prescribed medications	Life impact
Emotional wellbeing - emotional wellbeing includes lots of things like your mood, how often you worry, how often you get angry or upset, **how in control you feel**, your self esteem **and how you feel about the way others respond to you having diabetes.** *	Life impact
Fatigue - an overwhelming, sustained sense of exhaustion and decreased capacity for physical and mental work	Life impact
Global quality of life - someone's overall quality of life including physical, mental, and social wellbeing.	Life impact
Discontinuation or dose reduction - if someone is able to stop taking or reduce the dose of all or some of their diabetes medications	Life impact
Concomitant medication - how often and how many types of other medication someone has to take for example, blood pressure medication	Life impact
Perceived blood glucose control - how well someone with diabetes thinks their blood glucose is controlled	Life impact
Support - The support that is available to someone from their family, friends, peers and workplace	Life impact
Financial burden- The impact of someone's diabetes on their personal finances	Life impact
Diabetes self care activities- how well someone takes care of themselves in relation to their diabetes, for example, following dietary advice, foot care, testing and blood glucose levels	Life impact
Physical function - How well someone is able to function physically including how mobile they are and their physical strength.	Life impact
Sexual function - how well someone functions sexually	Life impact
Activities of daily living - being able to complete usual daily **activities including being able to drive.***	Life impact
Impact of diabetes on work or ability to work	Life impact
Being able to manage family responsibilities	Life impact
Social functioning - how able someone feels to join in social activities	Life impact
Cardiac function - how well the heart is working	Physiological/ clinical
Cardiorespiratory fitness - how well your respiratory and circulatory systems respond to exercise	Physiological/ clinical
Heart failure	Physiological/ clinical

Heart rate	Physiological/ clinical
Nonfatal myocardial infarction - having a heart attack that is not fatal	Physiological/ clinical
Retinopathy - damage to the blood vessels in the back of the eye caused by high blood glucose levels	Physiological/ clinical
Visual deterioration or blindness - if someone's eyesight gets worse or if they have loss of vision including blindness	Physiological/ clinical
Gastroparesis - this means that the stomach can't empty itself in the normal way. Symptoms can include feeling full/bloated, nausea/vomiting, loss of appetite, tummy pain/discomfort.	Physiological/ clinical
Exocrine pancreas function -how well your pancreas functions to make enzymes that help digest food	Physiological/ clinical
Body fat distribution - where body fat is stored in the body	Physiological/ clinical
Body weight - how much someone weighs	Physiological/ clinical
Liver function - how well the liver is working. The liver breaks down drugs and harmful substances. It also produces nutrients for the body to use including the storage and release of glucose.	Physiological/ clinical
Biomarkers of inflammation- Inflammation is the body's immune response to things like bacteria and viruses. The body can also sometimes attack its own tissues causing inflammation.	Physiological/ clinical
Having an infection in one or both feet including a foot ulcer, infection of the tissue	Physiological/ clinical
Having a bone infection (osteomyelitis). People with diabetes are more at risk of osteomyelitis especially if they have a foot ulcer.	Physiological/ clinical
Having gangrene or having an amputation of the leg, foot or toe	Physiological/ clinical
Genital fungal infection- Having genital thrush or a similar infection	Physiological/ clinical
Urinary tract infection - having an infection in the urinary tract, including bladder, urethra or kidneys.	Physiological/ clinical
Diabetic ketoacidosis- Diabetic ketoacidosis occurs if the body cannot produce enough insulin. It is a serious short term complication of diabetes which can result in coma or even death if it is not treated quickly.	Physiological/ clinical
Glycaemic control - how well someone's blood glucose is controlled.	Physiological/ clinical
Glycaemic variability - how much someone's blood glucose changes over the course of the day	Physiological/ clinical
Hyperglycaemia - how often someone has high blood glucose	Physiological/ clinical
Hyperosmolar hyperglycaemic state - a rare but serious and potentially life threatening complication of having very high blood glucose levels (often over 40mmol/L).	Physiological/ clinical
Hypoglycaemia - how often someone has low blood glucose levels.	Physiological/ clinical
Insulin sensitivity- how sensitive someone's body is to the effects of insulin	Physiological/ clinical
Insulin secretion - how well the body responds to a meal by producing insulin	Physiological/ clinical
Metabolic rate - The rate that the body uses energy	Physiological/ clinical
Appetite	Physiological/ clinical
Dietary intake - a measure of what someone has eaten	Physiological/ clinical
Neuropathy - damage to the nerves caused by high glucose. This can lead to tingling and pain or numbness in the feet or legs. It can also affect bowel control, stomach emptying and sexual function	Physiological/ clinical
General health	Physiological/ clinical
Kidney function - how well someone's kidneys are working	Physiological/ clinical
Blood Pressure	Physiological/ clinical
Cerebrovascular disease (including stroke, subarachnoid haemorrhage, transient ischaemic attack and vascular dementia	Physiological/ clinical
Peripheral vascular function - How well(veins and arteries) in the body (outside the heart) are working. Narrowing of these blood vessels, particularly in the legs, that can lead to pain,	Physiological/ clinical

gangrene and amputation.	
Risk of developing cardiovascular disease (including lips and lipoprotein markers of risk like cholesterol and triglycerides)	Physiological/ clinical
Gut microbiome - the type/number of bacteria in someone's digestive tract.†	Physiological/ clinical
All other costs of diabetes care apart from how often someone needs to see a healthcare professional.	Resource use
How often someone is admitted to hospital because of their diabetes.	Resource use
Healthcare resource utilisation - how often someone needs to see a healthcare professional	Resource use
The impact that having diabetes has on the time or finances of others	Resource use
*Text in bold reflects amendments to the outcome in R2 that were made in response to comments/feedback received in R1.	
† Outcome included in R2 only and added based on additional outcomes added by participants in R1	
