# Supplementary figures and images for: Selecting Core Outcomes for Randomised Effectiveness trials In Type 2 diabetes (SCORE-IT): a patient and healthcare professional consensus on a core outcome set for type 2 diabetes
Source: BMJ Open Diabetes Res Care. 2019 Dec 29;7(1):e000700. doi: 10.1136/bmjdrc-2019-000700 (PMC6936506; doi:10.1136/bmjdrc-2019-000700)

Supplementary document 9. Attrition bias between R1 and R2 by stakeholder group

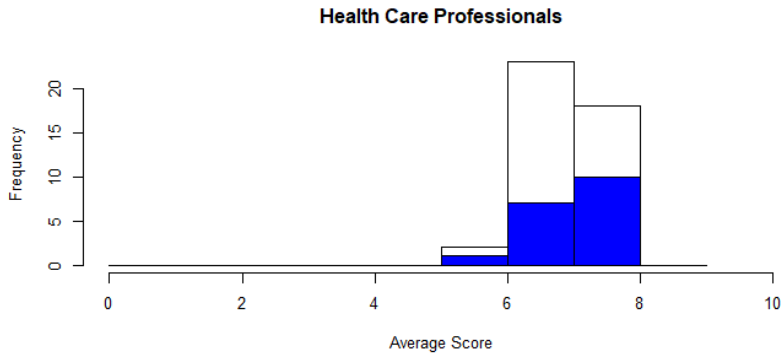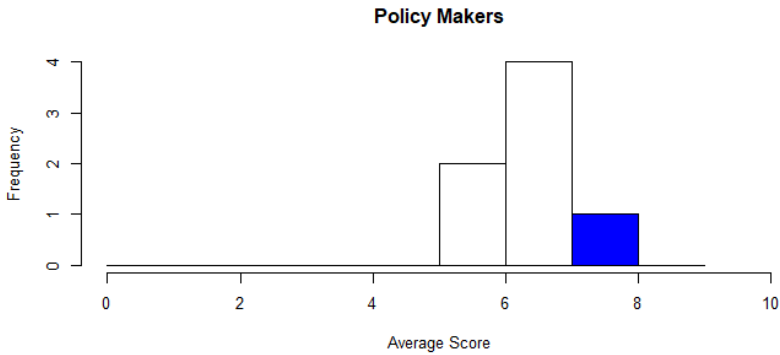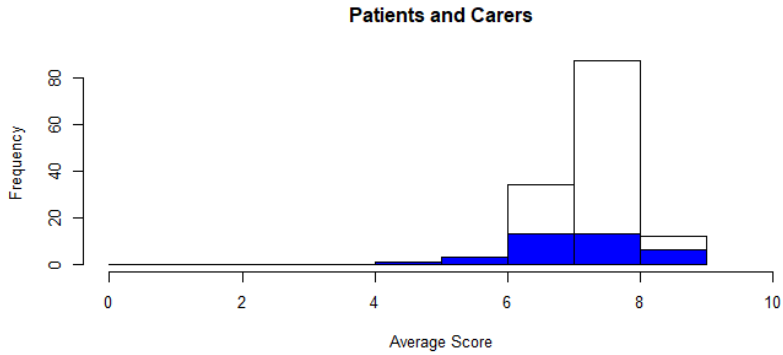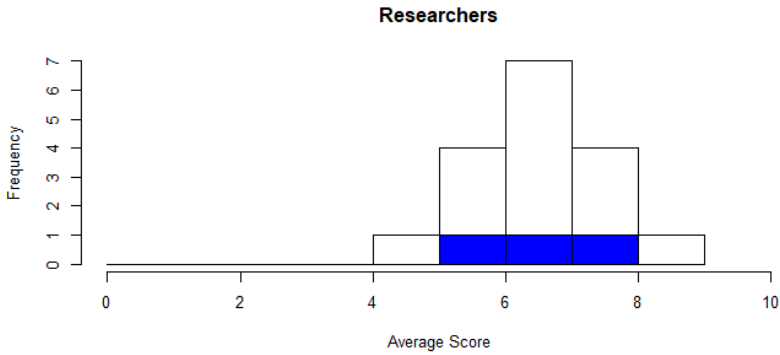

Supplement: Supplementary data [file bmjdrc-2019-000700supp005.pdf]
