# Supplementary material for: Selecting Core Outcomes for Randomised Effectiveness trials In Type 2 diabetes (SCORE-IT): a patient and healthcare professional consensus on a core outcome set for type 2 diabetes
Source: BMJ Open Diabetes Res Care. 2019 Dec 29;7(1):e000700. doi: 10.1136/bmjdrc-2019-000700 (PMC6936506; doi:10.1136/bmjdrc-2019-000700)
Supplement: Supplementary data [file bmjdrc-2019-000700supp006.pdf]

Selecting Core Outcomes for Randomised Effectiveness trials In Type 2 diabetes (SCORE-IT) – Consensus meeting report

Meeting date and time: 24th January, 10am-5pm

Location: University of Liverpool, UK

Table of Contents

1. Summary 1

   Outcomes included in the SCORE-IT core outcome set 2

2. Attendees 3
3. Pre Meeting 4
4. Meeting overview 4

   Voting and definition of consensus 5

5. Outcome discussion and voting. 5

   Outcomes session 1 – outcomes reaching the definition of consensus "in" 5

   Outcomes session 2. Outcome reaching the definition of consensus "out"........ 6

   Session 3. Outcomes with two or more groups reaching consensus "in" 7

   Session 4. Outcomes with one group scoring consensus "in" 18

6. Outcomes not discussed at the consensus meeting 20
7. Consensus meeting feedback........ 21

1. Summary

A meeting was held on the 24th January 2019 to review the results of the SCORE-IT study online Delphi and agree a core outcome set. The meeting was attended by: health professionals, people with type 2 diabetes, healthcare policy makers and observers.

Prior to the meeting the results of the second and final round of the online Delphi survey were analysed by stakeholder group and categorised according to a pre-defined definition of consensus. These results formed the basis of discussion and voting. For outcomes where all groups agreed either consensus "in" or consensus "out" there was no voting, outcomes with mixed results by stakeholder group were discussed and participants at the meeting asked to re-vote based on the discussion.

The meeting has resulted in a core outcome set for trials of glucose lowering interventions for type 2 diabetes. The next step, not described in this report, will involve identifying how best to measure these outcomes.

This report details the discussions at the meeting and describes how the meeting determined the outcomes that will be included in the core outcome set, these are:

Harman NL, *et al. BMJ Open Diab Res Care* 2019; 7:e000700. doi: 10.1136/bmjdrc-2019-000700

Outcomes included in the SCORE-IT core outcome set

Outcome	Point of inclusion in the core outcome set	Domain
Side effects of treatment- any unwanted effects of the treatment	Consensus meeting	Adverse events
Overall survival - how long someone lives	Delphi	Death
Death from a specific cause such as heart disease	Delphi	
Global quality of life - someone's overall quality of life including physical, mental and social wellbeing.	Consensus meeting	Life impact
Activities of daily living - being able to complete usual everyday tasks and activities including those related to personal care; house hold tasks or community based tasks.	Consensus meeting	
Heart failure	Delphi	Physiological/ clinical
Having gangrene or having an amputation of the leg; foot or toe	Delphi	
Diabetic ketoacidosis- Diabetic ketoacidosis occurs if the body cannot produce enough insulin. It is a serious short term complication of diabetes which can result in coma or even death if it is not treated quickly.	Delphi	
Hyperglycaemia - how often someone has high blood glucose	Delphi	
Glycaemic control - how well someone's blood glucose is controlled.	Consensus meeting	
Hypoglycaemia - how often someone has low blood glucose levels.	Delphi	
Cerebrovascular disease (including stroke; subarachnoid haemorrhage, transient ischaemic attack and vascular dementia	Delphi	
Nonfatal myocardial infarction - having a heart attack that is not fatal	Consensus meeting	
Visual deterioration or blindness - if someone's eyesight gets worse or if they have loss of vision including blindness	Consensus meeting	
Neuropathy - damage to the nerves caused by high glucose. This can lead to tingling and pain or numbness in the feet or legs. It can also affect bowel control; stomach emptying and sexual function	Consensus meeting	
Kidney function - how well someone's kidneys are working	Delphi	
Body weight - how much someone weighs	Consensus meeting	
How often someone is admitted to hospital because of their diabetes.	Delphi	Resource Use

Harman NL, *et al. BMJ Open Diab Res Care* 2019; 7:e000700. doi: 10.1136/bmjdrc-2019-000700

2. Attendees

Initials	Role	Stakeholder Group	Organisation/Country
**SB**	Independent Facilitator	N/A	Birmingham University, UK
**HB**	COMET Patient and Public Coordinator	N/A	University of Liverpool, UK
**PW**	Non-voting meeting contributor, Study Management Group, PI	N/A	University of Liverpool, UK
**NH**	Non-voting meeting contributor, Study Management Group	N/A	University of Liverpool, UK
**JD-M**	Observer, CORBEL study team	N/A	ECRIN, France
**AS-M**	Observer, CORBEL study team	N/A	German Research Centre for Environmental Health, Germany
**CP**	Observer, CORBEL study team	N/A	Netherlands
**SB**	Observer, CORBEL study team	N/A	ECRIN, France
**DC**	Meeting Participant, SSC member	Patient with type 2 diabetes	UK
**GT**	Meeting Participant, SSC member	Patient with type 2 diabetes	UK
**JL**	Meeting Participant, SSC member	Consultant	UK
**JH**	Meeting Participant, SSC member	Patient with type 2 diabetes	UK
**JP**	Meeting Participant, SSC member	Patient with type 2 diabetes	UK
**JW**	Meeting Participant, SSC chair	Consultant	University of Liverpool, UK
**ST**	Non-voting meeting Participant, Meeting Participant, SSC member	Healthcare Policy Maker	Centre for Medical Technology Policy, US
**CPO**	Meeting Participant	Health professional/researcher	University Putra Malaysia
**CBH**	Meeting Participant	Health professional/researcher	University Putra Malaysia /Universiteit Utrecht, Netherlands
**CS**	Meeting Participant	Patient with type 2 diabetes	UK
**DC**	Meeting Participant	Consultant	DiaCare, India
**EV**	Meeting Participant	Specialist Nurse	University of West Attica, Greece
**FR**	Meeting Participant	Family Physician	University of Buenos Aires, Argentina
**FE**	Meeting Participant	Patient with type 2 diabetes	UK
**JT**	Meeting Participant	Patient with type 2 diabetes	UK
**KR**	Meeting Participant	Patient with type 2 diabetes	UK
**KT**	Meeting Participant	Patient with type 2 diabetes	UK

**MO**	Meeting Participant	Healthcare Policy Maker	SBU Swedish Agency for Health Technology Assessment and Assessment of Social Services, Sweden
**NL**	Meeting Participant	Patient with type 2 diabetes	UK
**SB**	Meeting Participant	Patient with type 2 diabetes	UK
**SW**	Meeting Participant	Healthcare Policy Maker	SBU Swedish Agency for Health Technology Assessment and Assessment of Social Services, Sweden
**TM-R**	Meeting Participant	Patient with type 2 diabetes	UK
**HD**	Observer	N/A	University of Nottingham, UK
**AB**	Observer/note taker	N/A	University of Liverpool, UK
**KB**	Observer/note taker	N/A	University of Liverpool, UK
**NS**	Observer/note taker	N/A	University of Liverpool, UK

3. Meeting Participants

An invitation to attend the core outcome set consensus meeting was sent to participants of the online Delphi survey who had completed both rounds on the survey and who had expressed an interest in attending the consensus meeting.

All those who confirmed attendance received an email with information on what they should expect at the meeting, a copy of their scores from the online survey, a copy of the COMET lay summary, an information sheet outlining what would happen at the meeting, a meeting agenda and venue/travel information.

4. Pre-Meeting for Patients

A separate session was scheduled immediately before the main consensus meeting for 30 minutes to allow HB and SB to meet with patients attending the meeting. This meeting allowed patients to meet one another and for any questions to be answered about the structure of the day, expectations and for additional information to be given on core outcome set development methods.

5Meeting overview

The meeting started with an introduction from SB who welcomed everyone to the meeting and thanked them for attending and participating in the study. Following introductions from the meeting participants SB outlined the approach for the day, the role of the facilitator and the ground rules for the meeting. SB also stressed that she was not a health professional in the area of type 2 diabetes and that her role was an independent facilitator. SB advised that the meeting should focus on

"what" to measure and not to worry about "how". SB also asked participants to give consideration to what had already been included in the core outcome set (COS) when voting on an outcome and whether it is critical to include.

NH gave a brief overview of the study and what has happened to lead up to this point. During this presentation the scope of the core outcome set was clarified along with the definition of consensus that has been applied and used to generate the consensus matrix provided to participants by email and in hard copy at the meeting.

**Scope of the Core Outcome Set.**	
**Health Condition:**	Type 2 diabetes
**Population :**	Adults
**Intervention:**	Non-surgical therapeutic interventions for the treatment of hyperglycaemia
**Setting:**	Clinical trials

Voting and definition of consensus

A total of 20 meeting participants voted using turning point software and a 1-9 scoring scale (1, not important, 9 critical for inclusion in the core outcome set).

For the purpose of the consensus meeting, voting participants were grouped into healthcare professionals (n=7) and people with type 2 diabetes (n=13). Healthcare policy makers were present to contribute to the discussion and provided their scores separately.

Where there was voting on an outcome both groups were required to achieve the definition of consensus in i.e. 70% or more scoring 7-9.

Consensus classification	Description	Definition
Consensus in	Consensus that outcome should be included in the core outcome set	70% or more participants scoring as 7–9 AND < 15% participants scoring as 1–3
Consensus out	Consensus that outcome should not be included in the core outcomes set	50% or fewer participants scoring 7–9 in each stakeholder group.
No consensus	Uncertainty about importance of outcome	Anything else

4. Outcome discussion and voting

Outcomes session 1 – outcomes reaching the definition of consensus "in"

The meeting continued with a brief discussion of the outcomes that had reached "consensus in" in all stakeholder groups after the second round of the Delphi survey (9 outcomes). There was some

Harman NL, *et al. BMJ Open Diab Res Care* 2019; 7:e000700. doi: 10.1136/bmjdrc-2019-000700

discussion about hyperglycaemia and heart failure between patients and health professionals but all were happy for these to be included.

SB then asked how people at the meeting had interpreted “admission to hospital” and whether they had thought about it in terms of resource use at the hospital, the impact on their life or as indicator of the severity of their diabetes. There was discussion amongst the group which expressed a desire to avoid hospitalisation and the risk of infection from being in hospital, that hospitalisation had an impact on day to day life, that hospitalisation is often associated with increasing severity of type 2 diabetes and a cost to the health service, it was also noted that having type 2 diabetes could increase a hospital stay if admitted for another reason. Hospitalisation may also be captured as a complication or, in a trial of an investigational medicinal problem, as an adverse event. All agreed that although considered differently by different people “how often someone is admitted to hospital due to diabetes” should be included as an outcome.

Outcomes session 2 - outcomes reaching the definition of consensus “out”

The next session focused on outcomes that had met the definition of “consensus out” in all four stakeholder groups. There was no discussion for these outcomes with the exception of sexual function. The study team had flagged to SB that the gender of those completing the online survey was not recorded and that the ratio of males to females taking part in the survey may have influenced the results. SB specifically asked meeting participants about the exclusion of “sexual function”.

There was discussion that a decline in sexual function may be a side effect of treatment or may be related to neuropathy. There was also discussion about whether the COS needed to be considered for a specific age range and gender. NH reported that gender of the participants was not captured in the Delphi.

PW and SB also clarified that the COS discussed today should be for all adult patients with type 2 diabetes. The consideration of sub-groups of patients and the impact on outcomes reaching consensus is something that can be considered in the next steps of the project. PW clarified that the development of a COS is not a one off activity but instead the start of a process to improve research.

Harman NL, *et al. BMJ Open Diab Res Care* 2019; 7:e000700. doi: 10.1136/bmjdrc-2019-000700

Session 3 - outcomes with two or more groups reaching consensus" in"

Thirty two outcomes had no consensus in the Delphi but had at least one stakeholder group scoring the outcome as "consensus in". These were discussed in order of the outcomes with three groups reaching consensus in followed by those with two groups reaching consensus in.

Side effects of treatment

There was discussion about side effects of treatment, with health professionals explaining that it is critical to include this outcome as it is important to look at the risks and harms of a new treatment as well as its effectiveness. It was noted that experience of side effects might have impacted the score given and also that the term "side effects" was potentially trivialised by Delphi participants and did not cover the range of the "harms" outcome. There was also discussion about polypharmacy and whether the side effect was attributed to the trial treatment, a drug interaction or another treatment. It was clarified that all side effects should be considered and in a trial situation and that the protocol and randomisation procedures would ensure that issues of concomitant medication were addressed.

After hearing the discussion participants voted.

Result: consensus in

	Healthcare Professionals				People with type 2 diabetes			
	%1-3	%4-6	%7-9	Result	%1-3	%4-6	%7-9	Result
Side Effects of treatment	0	0	100	In	0	8	92	In

Death from a medical procedure

There was discussion around whether this outcome differed from the two death outcomes already included in the COS. Healthcare policymakers present commented that they had scored this low in the Delphi as they considered it to be covered by other outcomes. The decision was made to vote on whether this outcome added anything extra to the outcomes that were already included in the COS "overall survival" and "death from a specific cause such as heart disease"

Result: Consensus out

	Healthcare Professionals				People with type 2 diabetes			
	%1-3	%4-6	%7-9	Result	%1-3	%4-6	%7-9	Result
Death from a medical procedure	57	14	29	Out	38	46	15	Out

Harman NL, et al. BMJ Open Diab Res Care 2019; 7:e000700. doi: 10.1136/bmjdrc-2019-000700

Need for change in treatment or having to start taking a new or additional treatment because of high blood glucose.

There were some differences in interpretation of this outcome as it could be interpreted as treatment burden or as an indicator of worsening blood glucose control. Health professionals commented that if an intervention was not effective then additional treatments would be added and that these could also include rescue therapy for high blood glucose. There were comments from people with type 2 diabetes that this outcome should be considered critical as it indicates a treatment failure. There was also discussion about reporting in trials and that it would be important to report whether or not one treatment group needed more additional treatments than another.

Result: Consensus out

	**Healthcare Professionals**				**People with type 2 diabetes**			
	**%1-3**	**%4-6**	**%7-9**	**Result**	**%1-3**	**%4-6**	**%7-9**	**Result**
Need for change in treatment or having to start taking a new or additional treatment because of high blood glucose.	0	14	86	In	0	38	62	Out

Adherence to treatment - how well someone follows treatment instructions, for example taking all of their prescribed medications

There was discussion around whether adherence was an issue because someone was unable to adhere to the treatment or because they did not follow instructions. SB clarified that the outcome should be considered for any reason.

Further discussion followed about whether this represented a process outcome rather than an outcome of treatment.

PW re-iterated that when thinking about including outcomes in a COS you should consider the question “Do they affect your decision making between treatment A and treatment B”.

Harman NL, *et al. BMJ Open Diab Res Care* 2019; 7:e000700. doi: 10.1136/bmjdrc-2019-000700

Result: Consensus out

	Healthcare Professionals				People with type 2 diabetes			
	%1-3	%4-6	%7-9	Result	%1-3	%4-6	%7-9	Result
Adherence to treatment - how well someone follows treatment instructions for example; taking all of their prescribed medications	14	57	29	Out	15	69	15	Out

Global quality of life

People with type 2 diabetes expressed that this was an important outcome with emotive examples of how diabetes could impact on quality of life. One meeting participant stated *"from the perspective of patients, quality of life is the single most important thing to me".* One healthcare professional noted that overall quality of life is lower in people with type 2 diabetes compared to the general population and so when treating patients considers an improvement in quality of life to be one of the treatment goals. Healthcare policy makers commented that when reviewing the literature, quality of life is the measurement lacking from the majority of studies.

Result: Consensus in

	Healthcare Professionals				People with type 2 diabetes			
	%1-3	%4-6	%7-9	Result	%1-3	%4-6	%7-9	Result
Global quality of life - someone's overall quality of life including physical; mental; and social wellbeing.	0	14	86	In	0	0	100	In

Harman NL, *et al. BMJ Open Diab Res Care* 2019; 7:e000700. doi: 10.1136/bmjdrc-2019-000700

Cardiac function - how well the heart is working

Health professionals commented that cardiac function was an intermediate outcome used to assess heart failure (already included in the COS) and cardiovascular disease and that this does not need to be a separate core outcome but would be part of “heart failure”. Health professionals also went on to clarify that heart failure can be from a number of causes including myocardial infarction or damage to the heart muscle from hyperglycaemia.

Result: Consensus out

	Healthcare Professionals				People with type 2 diabetes			
	%1-3	%4-6	%7-9	Result	%1-3	%4-6	%7-9	Result
Cardiac function - how well the heart is working	29	57	14	Out	46	46	8	Out

Nonfatal myocardial infarction - having a heart attack that is not fatal

Health professionals commented that diabetes is considered as a disease of the heart, cardiovascular disease is important and myocardial infarction is one aspect of this. It was also clarified that heart failure and myocardial infarction are different. Patients asked if medication increased the risk of myocardial infarction, it was clarified that having type 2 diabetes increases the risk of having a myocardial infarction independently to any increased risks from medications.

Result: Consensus in

	Healthcare Professionals				People with type 2 diabetes			
	%1-3	%4-6	%7-9	Result	%1-3	%4-6	%7-9	Result
Nonfatal myocardial infarction - having a heart attack that is not fatal	14	14	71	In	0	8	92	In

Harman NL, *et al. BMJ Open Diab Res Care* 2019; 7:e000700. doi: 10.1136/bmjdrc-2019-000700

Retinopathy and Visual Deterioration

The group discussed that retinopathy is perhaps not an outcome but instead a measure of blood glucose control and also indicates the extent of microvascular problems. It is a core complication of diabetes but as few trials measure it, it is unknown if treatments improve retinopathy or not. It was also clarified that you can have retinopathy without vision problems.

All agreed to vote on the outcome "visual deterioration" before voting on "retinopathy".

Visual deterioration, result: Consensus in

	Healthcare Professionals				People with type 2 diabetes			
	%1-3	%4-6	%7-9	Result	%1-3	%4-6	%7-9	Result
Visual deterioration or blindness - if someone's eyesight gets worse or if they have loss of vision including blindness	0	14	86	In	0	0	100	In

Retinopathy, result: Consensus out

	Healthcare Professionals				People with type 2 diabetes			
	%1-3	%4-6	%7-9	Result	%1-3	%4-6	%7-9	Result
Retinopathy - damage to the blood vessels in the back of the eye caused by high blood glucose levels	0	43	57	Out	8	38	54	Out

Harman NL, *et al. BMJ Open Diab Res Care* 2019; 7:e000700. doi: 10.1136/bmjdrc-2019-000700

Having an infection in one or both feet including a foot ulcer; infection of the tissue

Having a bone infection (osteomyelitis). People with diabetes are more at risk of osteomyelitis especially if they have a foot ulcer.

It was suggested that the outcomes "having an infection in one or both feet…" and "having a bone infection (osteomyelitis)…" were discussed together as they were linked. Health professionals commented that you do not get osteomyelitis unless you have a foot ulcer/s. also that osteomyelitis can mean weeks or months in hospital as the minimum treatment is 6 weeks of intravenous antibiotics.

There was then further discussion about whether osteomyelitis was important to include in addition to "gangrene" which was already included. Patients also noted that some outcomes should be about keeping well rather than an end stage outcome. Discussions also took place that cellulitis or a foot ulcer could lead to septicaemia and death making it a significant outcome.

Agreed to vote on each outcome and if included then to have a discussion about how to group them afterwards.

Having an infection in one or both feet including a foot ulcer; infection of the tissue

Result: Consensus out

	Healthcare Professionals				People with type 2 diabetes			
	**%1-3**	**%4-6**	**%7-9**	**Result**	**%1-3**	**%4-6**	**%7-9**	**Result**
Having an infection in one or both feet including a foot ulcer; infection of the tissue	0	43	57	Out	0	8	92	In

Having a bone infection (osteomyelitis). People with diabetes are more at risk of osteomyelitis especially if they have a foot ulcer.

Result: Consensus out

	Healthcare Professionals				People with type 2 diabetes			
	**%1-3**	**%4-6**	**%7-9**	**Result**	**%1-3**	**%4-6**	**%7-9**	**Result**
Having a bone infection (osteomyelitis). People with diabetes are more at risk of osteomyelitis especially if they have a foot ulcer.	0	43	57	Out	8	23	69	Out

Harman NL, *et al. BMJ Open Diab Res Care* 2019; 7:e000700. doi: 10.1136/bmjdrc-2019-000700

Glycaemic control - how well someone's blood glucose is controlled

Health professionals explained that this outcome refers to an overall measure of glycaemic control; in clinical practice this is routinely assessed by measuring HbA1c. Policymakers commented that there is a preference to measure hard outcomes and complications rather than intermediate ones like HbA1c. There was then further discussion that it was important to include glycaemic control as an efficacy measure. It was also noted that hyperglycaemia and glycaemic control could be combined as an outcome.

Result: Consensus in

	Healthcare Professionals				People with type 2 diabetes			
	%1-3	%4-6	%7-9	Result	%1-3	%4-6	%7-9	Result
Glycaemic control - how well someone's blood glucose is controlled.	0	0	100	In	0	0	100	In

Hyperosmolar hyperglycaemic state - a rare but serious and potentially life threatening complication of having very high blood glucose levels (often over 40mmol/L).

Health professionals commented that hyperosmolar hyperglycaemic state, ketoacidosis are acute metabolic emergencies. Patients are very unwell with very high glucose and are dehydrated. In 100% of cases patients will be admitted to hospital. There was discussion that if voted in this would be included under "hyperglycaemic emergencies" with ketoacidosis.

Result: Consensus out

	Healthcare Professionals				People with type 2 diabetes			
	%1-3	%4-6	%7-9	Result	%1-3	%4-6	%7-9	Result
Hyperosmolar hyperglycaemic state - a rare but serious and potentially life threatening complication of having very high blood glucose levels (often over 40mmol/L).	0	29	71	In	8	23	69	Out

Harman NL, *et al. BMJ Open Diab Res Care* 2019; 7:e000700. doi: 10.1136/bmjdrc-2019-000700

Neuropathy - damage to the nerves caused by high glucose. This can lead to tingling and pain or numbness in the feet or legs. It can also affect bowel control; stomach emptying and sexual function

SB reminded participants that neuropathy included sexual function which was currently out. Patients expressed that they thought neuropathy was an important outcome that was often overlooked until more severe and also discussed the impact on quality of life and on daily tasks such as using a keyboard and driving. Health professionals explained that neuropathy was a spectrum in terms of severity. SB reminded everyone that if neuropathy is important it should be voted in even if some aspects might be captured by global quality of life.

Result: Consensus in

	Healthcare Professionals				People with type 2 diabetes			
	%1-3	%4-6	%7-9	Result	%1-3	%4-6	%7-9	Result
Neuropathy - damage to the nerves caused by high glucose. This can lead to tingling and pain or numbness in the feet or legs. It can also affect bowel control; stomach emptying and sexual function	14	14	71	In	0	8	92	In

Kidney function - how well someone's kidneys are working

Health professionals explained that kidney function was part of routine assessments, kidney failure is high in people with type 2 diabetes and kidney function can also be affected by some medications.

Result: Consensus in

	Healthcare Professionals				People with type 2 diabetes			
	%1-3	%4-6	%7-9	Result	%1-3	%4-6	%7-9	Result
Kidney function - how well someone's kidneys are working	0	0	100	In	0	8	92	In

Harman NL, *et al. BMJ Open Diab Res Care* 2019; 7:e000700. doi: 10.1136/bmjdrc-2019-000700

Blood pressure

Health professionals discussed that blood pressure and "risk of developing cardiovascular disease" are risk factors for cerebrovascular disease, heart failure and myocardial infarction that are already included. They also commented that as a risk factor blood pressure is as important as blood glucose. SB asked the question "is blood pressure something that would critically inform decision making for a treatment or are the later stage risks more important?"

Result: Consensus out

	Healthcare Professionals				People with type 2 diabetes			
	%1-3	%4-6	%7-9	Result	%1-3	%4-6	%7-9	Result
Blood Pressure	14	14	71	In	0	54	46	Out

Risk of developing cardiovascular disease (including lipid and lipoprotein markers of risk like cholesterol and triglycerides)

No further discussion prior to voting.

Result: Consensus out

	Healthcare Professionals				People with type 2 diabetes			
	%1-3	%4-6	%7-9	Result	%1-3	%4-6	%7-9	Result
Risk of developing cardiovascular disease (including lipid and lipoprotein markers of risk like cholesterol and triglycerides)	14	29	57	Out	15	54	31	Out

Harman NL, *et al. BMJ Open Diab Res Care* 2019; 7:e000700. doi: 10.1136/bmjdrc-2019-000700

Activities of daily living - being able to complete usual everyday tasks and activities including those related to personal care; house hold tasks or community based tasks.

Patients expressed that activities of daily living were a critical part of overall quality of life, patients were also conscious that life impact outcomes had not been included and so would feel uncomfortable if this did not go in as so far the outcomes are not patient reported and therefore patients have a passive role in the outcomes. There was also some discussion from health professionals that physical and cognitive function were needed for activities of daily living.

There was an initial vote where, despite there being discussion around this outcome being critical, the outcome was voted out. SB queried why the argument for this outcome not being important had not been put across and then asked for input from those who had voted important but not critical.

One health professional commented that people with type 2 diabetes do not have issues with daily living. However, another health professional expressed that the impact on activities of daily living depends on disease severity and so cannot be based solely on people seen in a specific clinic. Others (patients and health professionals) explained they had voted with a score of 6 as it was important but not important enough to include in all studies or that other outcomes cover issues that would impact on activities of daily living. Also that if other outcomes were treated/resolved then activities of daily living would not be a concern. Conversely patients commented that activities of daily living are more important than clinicians realise and asked health professionals to think about how they would score the outcome if it affected them.

Result: Consensus in

	**Healthcare Professionals**				**People with type 2 diabetes**			
	**%1-3**	**%4-6**	**%7-9**	**Result**	**%1-3**	**%4-6**	**%7-9**	**Result**
Activities of daily living - being able to complete usual everyday tasks and activities including those related to personal care; house hold tasks or community based tasks.	0	14	86	In	0	8	92	In

Harman NL, *et al*. *BMJ Open Diab Res Care* 2019; 7:e000700. doi: 10.1136/bmjdrc-2019-000700

Peripheral vascular function - How well (veins and arteries) in the body (outside the heart) are working. Narrowing of these blood vessels, particularly in the legs, can lead to pain, gangrene and amputation.

No arguments for this being a critical outcome were put forward prior to voting.

Result: Consensus out

	Healthcare Professionals				People with type 2 diabetes			
	%1-3	%4-6	%7-9	Result	%1-3	%4-6	%7-9	Result
Peripheral vascular function - How well (veins and arteries) in the body (outside the heart) are working. Narrowing of these blood vessels, particularly in the legs, can lead to pain, gangrene and amputation.	14	71	14	Out	15	54	31	Out

Gastroparesis

Only one comment was made from a healthcare professional that gastroparesis is included in the neuropathy outcome.

Result: Consensus out

	Healthcare Professionals				People with type 2 diabetes			
	%1-3	%4-6	%7-9	Result	%1-3	%4-6	%7-9	Result
Gastroparesis - this means that the stomach cannot empty itself in the normal way. Symptoms can include feeling full/bloated; nausea/vomiting, loss of appetite, tummy pain/discomfort.	0	86	14	Out	8	77	15	Out

Session 4 - outcomes with one group scoring "consensus in"

At this point SB presented the outcomes where only one group had reached the definition of consensus in after the second round of the Delphi. Due to time constraints SB asked which on the list, if any, participants would like to discuss.

Four outcomes were put forward as needing discussion:

- **Body weight**
- **Cognitive function**
- **Diabetes self-management activities**
- **Insulin sensitivity**

Body weight

Health professionals put forward that increased body weight predicts poor outcomes for people with type 2 diabetes, also that some treatments might make body weight better or worse. There was also a comment that body weight can be managed by patients and is therefore empowering to people with type 2 diabetes.

Result: Consensus in

	**Healthcare Professionals**				**People with type 2 diabetes**			
	**%1-3**	**%4-6**	**%7-9**	**Result**	**%1-3**	**%4-6**	**%7-9**	**Result**
Body weight - how much someone weighs	0	29	71	In	0	15	85	In

Cognitive function - things about someone's memory, concentration, language, thinking and ability to understand instructions

Patients put forward that cognitive function affects decision making and how well someone can manage their diabetes. Also that the Diabetes UK clinical studies group recognises cognitive function and mental health as important. It was clarified that in terms of outcomes, mental health and cognitive function are different things. Dementia may be more common in people with type 2 diabetes but the evidence is not clear. Cognitive function was also discussed in the context of hypoglycaemia and also how cognitive function can be difficult to define culturally.

Harman NL, *et al. BMJ Open Diab Res Care* 2019; 7:e000700. doi: 10.1136/bmjdrc-2019-000700

Result: Consensus out

	Healthcare Professionals				People with type 2 diabetes			
	%1-3	%4-6	%7-9	Result	%1-3	%4-6	%7-9	Result
Cognitive function - things about someone's memory, concentration, language, thinking and ability to understand instructions.	14	71	14	Out	8	54	38	Out

Diabetes self-care activities- how well someone takes care of themselves in relation to their diabetes; for example; following dietary advice; foot care; testing and blood glucose levels

Patients put forward that the level of support given varies and that this could influence how well someone manages their diabetes. Healthcare professionals felt that this was not an outcome but an issue of service delivery. It was noted that adherence was voted out which would be similar to this outcome in terms of how well someone can follow self-care advice.

Result: Consensus out

	Healthcare Professionals				People with type 2 diabetes			
	%1-3	%4-6	%7-9	Result	%1-3	%4-6	%7-9	Result
Diabetes self-care activities- how well someone takes care of themselves in relation to their diabetes; for example; following dietary advice; foot care; testing and blood glucose levels	14	43	57	Out	8	54	38	Out

Insulin sensitivity- how sensitive someone's body is to the effects of insulin

Patients put forward that increased insulin resistance (lower insulin sensitivity) results in increased blood glucose. Health professionals noted that, whilst insulin resistance is a risk factor, once identified it does not change that much and also that all participants in a trial who have type 2 diabetes will have insulin resistance.

Result: Consensus out

	Healthcare Professionals				People with type 2 diabetes			
	**%1-3**	**%4-6**	**%7-9**	**Result**	**%1-3**	**%4-6**	**%7-9**	**Result**
Insulin sensitivity- how sensitive someone's body is to the effects of insulin	29	71	0	Out	25	58	17	Out

5. Outcomes not discussed at the consensus meeting

Outcomes where no group had reached the definition of consensus (n=15) in were not discussed at the consensus meeting. These were:

Satisfaction with treatment and care - how satisfied someone with diabetes is with the treatments they are taking/following, the care that they receive from healthcare professionals and the amount and type of diabetes information available
Emotional wellbeing - emotional wellbeing includes lots of things like someone's mood, how often they worry, feel anxious or sad, how often they get angry or upset and their self-esteem.
Fatigue - an overwhelming; sustained feeling of exhaustion, mental or physical tiredness, having little energy for physical and mental work.
Concomitant medication - how often and how many types of other medication someone has to take for example; blood pressure medication
Perceived blood glucose control - how well someone with diabetes thinks their blood glucose is controlled
Financial burden- The impact of someone's diabetes on their personal finances
Impact of diabetes on work or ability to work
Being able to manage family responsibilities
Social functioning - how able someone feels to join in social activities and maintain relationships with others.
Heart rate
Biomarkers of inflammation- Inflammation is the body’s immune response to things like bacteria and viruses. The body can also sometimes attack its own tissues causing inflammation.
Genital fungal infection- Having genital thrush or a similar infection
Urinary tract infection - having an infection in the urinary tract, including bladder; urethra or kidneys.
General health
Healthcare resource utilisation - how often someone needs to see a healthcare professional

Harman NL, *et al. BMJ Open Diab Res Care* 2019; 7:e000700. doi: 10.1136/bmjdrc-2019-000700

6. Consensus meeting feedback

Feedback forms were provided to meeting participants and returned to NH at the end of the day. Nineteen feedback forms were received (people with type 2 diabetes n=12, other n=3, healthcare professionals n= 4). Responses to each question were scored from 1 (Strongly disagree) to 5 (strongly agree).

The feedback received is summarised below:

	Overall average (range)	Patient average	Healthcare professional average	Other average
**The information that the organisers provided in advance of the meeting was helpful**	**5 (3-5)**	5	5	5
**I was satisfied with the process used to agree core outcomes**	**4 (3-5)**	5	5	4
**I was satisfied with the way the meeting was facilitated**	**5 (4-5)**	5	5	5
**I felt able to contribute to the meeting**	**5 (4-5)**	5	5	4
**I felt comfortable in communicating my views**	**5 (4-5)**	5	5	5
**The workshop produced a fair result**	**4 (2-5)**	4	4	4

Free text feedback

Some concerns were raised about a dominant panellist, the impact of who spoke first (healthcare professional vs patient or male vs female).

Feedback also included the desire for a longer information session to discuss terminology, the impact of scoring less than 7 i.e. that the outcome would not be included and also whether there was some guidance that could be given on how many outcomes should be included in a core outcome set.

Feedback about the meeting location was positive however there was some feedback that a slightly larger room would have been better plus there were some issues with the air conditioning.

Harman NL, *et al. BMJ Open Diab Res Care* 2019; 7:e000700. doi: 10.1136/bmjdrc-2019-000700
