# Supplementary material for: Selecting Core Outcomes for Randomised Effectiveness trials In Type 2 diabetes (SCORE-IT): a patient and healthcare professional consensus on a core outcome set for type 2 diabetes
Source: BMJ Open Diabetes Res Care. 2019 Dec 29;7(1):e000700. doi: 10.1136/bmjdrc-2019-000700 (PMC6936506; doi:10.1136/bmjdrc-2019-000700)
Supplement: Supplementary data [file bmjdrc-2019-000700supp007.pdf]

SCORE-IT Study Steering Committee Meeting
Minutes
22nd March 2019

In attendance:
Paula Williamson (PW), John Wilding,(JW), Dave Curry (DC), Nic Harman (NH, minutes), Jennifer Logue (JL), Gareth Thompson (GT), Sean Tunis (ST).

Apologies received
Jim Harris (JH), John Pemberton (JP), Leigh Perreault (LP)

1. Discussion of core outcome set and final amendments:

All agreed that the outcomes “death from a specific cause” and “overall survival” represented separate outcome.

The group discussed the outcome “death from a specific cause such as heart disease” and that this was likely to have been interpreted as a diabetes related cause in the Delphi as the help text specified “cause specific mortality. All agreed to amend to “death from a diabetes related cause such as heart disease”.

Action: NH to amend to “death from a diabetes related cause such as heart disease”

NH presented the consensus meeting results for “hyperosmolar hyperglycaemic state”.

	**Healthcare Professionals**				**People with type 2 diabetes**			
	**%1-3**	**%4-6**	**%7-9**	**Result**	**%1-3**	**%4-6**	**%7-9**	**Result**
Hyperosmolar hyperglycaemic state - a rare but serious and potentially life threatening complication of having very high blood glucose levels (often over 40mmol/L).	0	29	71	In	8	23	69	Out

The SSC discussed that this was actually more common in people with type 2 diabetes than diabetic ketoacidosis and so it would be appropriate to include this outcome in a revised outcome called “hyperglycaemic emergencies”. All agreed, LP had sent email agreement on the 12-2-19.

Harman NL, *et al. BMJ Open Diab Res Care* 2019; 7:e000700. doi: 10.1136/bmjdrc-2019-000700

Action: NH to amend outcome to "hyperglycaemic emergencies – to include diabetic ketoacidosis and hyperosmolar hyperglycaemic state)

There was discussion around the outcome "hyperglycaemia" and "glycaemic control" and that whilst they are similar they were scored separately in the Delphi with "hyperglycaemia" reaching consensus after round 2 of the Delphi and "glycaemic control" being added to the core outcome set at the consensus meeting. There was also discussion about how hyperglycaemic may be complex to assess but that this aspect would be addressed at the "how" stage of the project.

All agreed that these should be retained as separate outcomes but that the study report should note a limitation in that this outcome would have benefited from further discussion prior to the launch of the Delphi as it's unclear how Delphi participants have interpreted the outcome.

Decision : "hyperglycaemia" and "glycaemic control" to be retained as separate outcomes.

Prolongation of hospital stay was discussed. This had been raised in the consensus meeting in the context of someone being admitted to hospital for an unrelated reason but then having their hospital stay prolonged because of their diabetes.

All agreed that "prolongation of hospital stay" was a separate outcome that had not been scored in the Delphi nor added a by a Delphi participant in round 1. The SCORE-IT study will report that this was raised at the consensus meeting and that it is a future point for discussion.

Action: NH to update SCORE-IT report

Harman NL, *et al. BMJ Open Diab Res Care* 2019; 7:e000700. doi: 10.1136/bmjdrc-2019-000700
